# Effects of St John's Wort (*Hypericum perforatum* L*.*) Extracts on Epileptogenesis

**DOI:** 10.3390/molecules16098062

**Published:** 2011-09-19

**Authors:** Vesna Ivetic, Svetlana Trivic, Marija Knezevic Pogancev, Mira Popovic, Janka Zlinská

**Affiliations:** 1 Laboratory of Neurophysiology, Department of Physiology, Medical Faculty, University of Novi Sad, Hajduk Veljkova 3, 21000 Novi Sad, Serbia; Email: godipo@eunet.rs; 2 Department of Chemistry, Faculty of Sciences, University of Novi Sad, Trg Dositeja Obradovica 3, 21000 Novi Sad, Serbia; Email: svetlana.trivic@dh.uns.ac.rs (S.T.); mira.popovic@dh.uns.ac.rs (M.P.); 3 University of Central Europe in Skalica, Královská 386/11, Skalica, 909 01 Trnavsky kraj, Slovakia; Email: j.zlinska@sevs.sk

**Keywords:** St. John's wort, *Hypericum perforatum* L., extracts, kindling, epilepsy, rabbits

## Abstract

The purpose of this study was to investigate the effects of treatment with water, *n*-butanol and ether extracts of *Hypercom perforatum* L*.* on epileptogenesis in rabbits. Animals from the control group received solvent-ethanol, and the kindling model of epilepsy was used. Epileptic focus was induced in Chinchilla rabbits by stimulation of the hippocampus. The following parameters were determined: the minimum current strength necessary to induce after-discharge (AD) – discharges appearing after cessation of stimulation; AD duration; the number of stimulations necessary to induce spontaneous kindling; and the latency time for the development of full kindling. The results obtained indicate that epileptogenesis is influenced by *Hypericum perforatum* L. extract treatment.Animals treated with an ether extract of *Hypericum perforatum* L. required significantly weaker minimum current strengths for the development of epileptogenic focus, and displayed longer AD times, while the number of electro-stimulations necessary for full kindling was less. In contrast, animals treated with water and *n*-butanol extracts required increased electro-stimulations for the development of epileptic discharge, and displayed shortened AD durations *versus* controls.

## 1. Introduction

The use of complementary alternative medicines, such as herbal-based medicines, is becoming increasingly popular [1,2,3]. The most commonly used herbal medicines are St John’s wort, echinacea, garlic, ginseng, ginkgo biloba, valerian, ephedra and kava [4]. 

In recent years, use of St. John's wort (*Hypericum perforatum* L.) for the treatment of premenstrual syndrome [5,6], as well as for problems associated with menopause, such as mood shifts, sweating, anxiety, redness of the face and insomnia [7,8,9] has been increasing. In addition, standardized extracts of *Hypericum perforatum* L. are now commonly used for the treatment of depressive disorders [10,11]. 

Results from early clinical studies have been mixed, with some showing beneficial effects and others suggesting no benefit from St. John's wort for the treatment of mild to moderate forms of depression [11,12]. However, there is evidence that St. John's wort extracts are more effective than placebo for the treatment of mild to moderately severe depressive disorders [13]. 

More recently, detailed studies have been conducted comparing the effects of different extracts of *Hypericum* *perforatum* L*. versus* standard antidepressives, using well-defined patient groups over longer periods of time [14]. Based on these studies, *Hypericum extracts* L. are significantly superior to placebo, and as effective as standard tricyclic antidepressive drugs. In addition, side effects were found to occur less often in *Hypericum* treated patients *versus* patients administered standard antidepressants [14,15]. The curative action of *Hypericum perforatum* L. is associated with the presence of a series of bioactive compounds. Extracts from the flowers and leaves of *Hypericum perforatum* L. contain at least ten classes of pharmacologically active components: flavonol derivatives, biflavones, proanthocyanidines, xanthones, phloroglucinols and naphthodianthrones [16,17]. Of these, hypericin and hyperforin [18,19] are the most studied. Both molecules are strongly active in biological systems [3,20], where they display an indirect influence on transmitter systems, manifest as changes in neuron excitability. The excitability of a neuron is determined by its membrane characteristics (including its various structures and canal receptors), along with system messengers (e.g., ion type and concentration, and various neurotransmitters), which generate effects through chemical synapses or ephaptic transfer.

It is widely accepted that epileptogenesis involves the transformation of normal, functional populations of neurons into chronically excited neurons, leading to hyper-synchronic activity in the neuron population [21]. Hyper-excitability is a manifestation of neuronal dysfunction, which can be caused by many, often unknown, factors. The associated increase in neural activity causes changes in the local ionic environment, which further changes the excitability of the neuronal population. Because the bioactive constituents of *Hypericum perforatum* L. have been shown to alter the excitability of neurons, in the present study we investigated the potential influence of various *Hypericum perforatim* L. extracts on epileptogenesis.

Although different animal models have been proposed to evaluate the process of epileptogenesis [22], the kindling model of epilepsy was chosen for the present study. This was because the kindling model, first described by Goddard approximately 40 years ago [23], is still one of the most often cited experimental models of epilepsy [24,25].

## 2. Results and Discussion

Values observed for the minimum current strength necessary for threshold after-discharge in each experimental group are presented in Table 1. In the control group, the minimum current strength necessary for threshold after-discharge was found to be 130 ± 18.70 μA, *versus* 140 ± 9.27 μA and 140.00 ± 15.36 μA in experimental groups B and W, while the lowest values were observed for group E (110 ± 11.66 μA). 

**Table 1:** Minimum current strength (μA) necessary for threshold after-discharge: summary statistics.

GROUP\VARIABLE	C	W	B	E
Sample size	9	9	9	9
Lowest value	130.00	140.00	140.00	110.00
Highest value	180.00	190.00	170.00	150.00
Arithmetic mean	150.00	168.88	161.11	131.11
95% CI for the mean	135.61 to 164.38	157.07 to 180.70	153.97 to 168.24	122.14 to 140.07
Median	150.00	170.00	160.00	130.00
95% CI for the median	130.00 to 168.62	152.75 to 180.00	160.00 to 170.00	121.38 to 140.00
Variance	350.00	236.11	86.11	136.11
Standard deviation	18.70	15.36	9.27	11.66
Relative standard deviation	0.12 (12.47%)	0.09 (9.10%)	0.05 (5.76%)	0.08 (8.90%)
Standard error of the mean	6.23	5.12	3.09	3.88
Coefficient of Skewness	0.29 (p = 0.6733)	−0.82 (p = 0.2411)	−1.46 (p = 0.0424)	0.26 (p = 0.7018)
Coefficient of Kurtosis	−1.33 (p =0.2981)	0.48 (p = 0.5940)	3.28 (p = 0.0556)	0.54 (p = 0.5695)
Kolmogorov-Smirnov test for Normal distribution	accept normality (p = 0.86)	accept normality (p = 0.70)	accept normality (p = 0.99)	accept norrmality (p = 0.61)

C - control group animals treated with solvent (50% ethanol); W - animals treated with the water fraction of a crude *Hypericum* extract; B - animals treated with the butanol fraction of a *Hypericum* extract; E - animals treated with the ether fraction of a crude *Hypericum* extract.

Correlation coefficients between the average values of minimum current strength in different groups are presented in Table 2, while the statistical difference between mean values of discharged current strength are shown in Table 3. 

**Table 2:** Pearson correlation coefficients between the average values of minimum current strength for different groups.

GROUP		C	W	B	E
C	Correlation Coefficient		−0.47	0.14	0.20
	Significance Level P		0.19	0.71	0.46
	n		9	9	9
W	Correlation Coefficient	−0.47		0.010.989	−0.20
	Significance Level P	0.19			0.60
	n	9			9
B	Correlation Coefficient	0.14	0.010.989		**0.68**
	Significance Level P	0.71			**0.04**
	n	9			9
E	Correlation Coefficient	0.28	−0.20	**0.68**	
	Significance Level P	0.45	0.60	**0.04**	
	n	9	9	9	

**Table 3:** Statistical difference between mean discharged current strength values *.

		C	W	B	E
****C****	Difference		18.89	11.11	−18.89
	Standard Error		8.07	6.96	7.35
	Test statistic t		2.341	1.596	−2.570
	Significance Level p		****p = 0.0325****	p = 0.136	****p = 0.0233****
****W****	Difference	18.89		7.78	37.78
	Standard Error	8.07		5.98	6.43
	Test statistic t	2.341		−1.300	−5.874
	Significance Level p	****p = 0.0325****		p = 0.2121	****p < 0.0001****
****B****	Difference	11.11	7.78		30.00
	Standard Error	6.96	5.98		4.97
	Test statistic t	1.596	−1.300		6.037
	Significance Level p	p = 0.136	p= 0.2121		****p < 0.0001****
****E****	Difference	−18.89	−37.78	30.00	
	Standard Error	7.35	6.43	4.97	
	Test statistic t	−2.570	−5.874	6.037	
	Significance Level p	****p = 0.0233****	****p < 0.0001****	****p < 0.0001****	

* using an independent samples t-test and assuming unequal variances.

As can be seen, significant statistical differences were observed between groups W and E, and between groups B to E (see Table 3).

As shown in Table 4, the discharge duration AD lasted for an average of 9.55 ± 2.50 s in the control group, and only 8.00 ± 1.41 s in the W group, and 8.33 ± 2.69 s in the group B. In contrast, group E displayed the longest AD duration (11.22 ± 1.56 s). Following a similar trend, the shortest discharge duration time AD observed in control, W and B group animals was 5 s, while for animals from group E, a duration time of 9s was recorded. The longest duration time for the first AD in both the control group and group E was 13 s.

**Table 4:** Threshold after-discharge duration times (in seconds) - summary statistics.

GROUP\VARIABLE	C	W	B	E
Sample size	9	9	9	9
Lowest value	5.00	5.00	5.00	9.00
Highest value	13.00	9.00	12.00	13.00
Arithmetic mean	9.55	8.00	8.33	11.22
95% CI for the mean	7.62 to 11.48	6.91 to 9.08	6.26 to 10.40	10.02 to 12.42
Median	10.00	9.00	8.00	12.00
95% CI for the median	8.00 to 11.86	7.00 to 9.00	5.13 to 11.72	9.13 to 12.86
Variance	6.27	2.00	7.250	2.44
Standard deviation	2.50	1.41	2.69	1.56
Relative standard deviation	0.26 (26.22%)	0.17 (17.68%)	0.32 (32.31%)	0.13 (13.93%)
Standard error of the mean	0.83	0.47	0.89	0.52
Coefficient of Skewness	−0.45 (p = 0.518)	−1.36 (p = 0.058)	0.13 (p = 0.844)	−0.46 (p = 0.503)
Coefficient of Kurtosis	−0.26 (p = 0.7024)	1.32 (p = 0.3016)	−1.27 (p = 0.3124)	−1.34 (p = 0.296)
Kolmogorov-Smirnov test for Normal distribution	accept normality (p = 0.9135)	accept normality (p = 0.8733)	accept normality (p = 0.9895)	accept normality (p = 0.7821)

Correlation coefficients for duration after discharge values observed for different groups are presented in Table 5. 

**Table 5:** Pearson correlation coefficients for duration after discharge values observed for different groups.

		C	W	B	E
**C**	Correlation Coefficient		0.85	0.14	0.22
	Significance Level P		**0.0040**	0.7274	0.5698
	n		9	9	9
**W**	Correlation Coefficient	0.85		0.56	0.51
	Significance Level P	**0.0040**		0.1184	0.1619
	n	9		9	9
**B**	Correlation Coefficient	0.14	0.56		0.43
	Significance Level P	0.7274	0.1184		0.2534
	n	9	9		9
**E**	Correlation Coefficient	0.22	0.51	0.43	
	Significance Level P	0.5698	0.1619	0.2534	
	n	9	9	9	

Comparison of the duration of threshold AD in the analyzed groups revealed that the average discharge duration AD in group E was significantly longer than in animals from group W and group B (see Table 6). In addition, analysis of the duration of threshold AD showed that the average discharge duration AD in group W was shorter than that observed for groups B and C, although these results were not significant.

**Table 6:** Statistical differences between mean values of threshold after-discharge current strength *.

		C	W	B	E
**C**	Difference		−1.56	−1.22	1.67
	Standard Error 95% CI of difference		0.96	1.23	0.98
	Test statistic t		−3.627 to 0.516	−3.821 to 1.3768	−0.460 to 3.793
			−1.622	−0.997	1.693
	Significance Level p			p = 0.3336	p = 0.1143
			p = 0.1288		
**W**	Difference	−1.56		0.33	3.22
	Standard Error	0.96		1.01	0.70
	95% CI of differenceTest statistic t	−3.62 to 0.516		−1.875 to 2.542	1.732 to 4.711
	Significance Level p	−1.622		0.329	4.585
		p = 0.1288		p = 0.7480	p = 0.0003
**B**	Difference	−1.22	0.33		2.89
	Standard Error	1.23	1.01		1.04
	95% CI of differenceTest statistic t	−3.821 to 1.37	−1.875 to 2.542		0.646 to 5.131
	Significance Level p	−0.997	0.329		2.783
		p = 0.3336	p = 0.7480		p = 0.0155
**E**	Difference	1.67	3.22	2.89	
	Standard Error	0.98	0.70	1.04	
	95% CI of differenceTest statistic t	−0.46 to 3.793	1.73 to 4.7119	0.646 to 5.131	
	Significance Level p	1.693	4.585	2.783	
		p = 0.1143	p = 0.0003	p = 0.0155	

* using an independent samples t-test and assuming unequal variances.

The average number of electro-stimulations necessary for the development of full kindling is shown in Table 7.

**Table 7:** The average number of electro-stimulations necessary for the development of full kindling - summary statistics.

GROUP\VARIABLE	C	W	B	E
Sample size	9	9	9	9
Lowest value	35.00	33.00	32.00	28.00
Highest value	47.00	49.0	47.0	39.0
Arithmetic mean	41.66	41.88	41.55	34.66
95% CI for the mean	38.47 to 44.85	37.81 to 45.96	37.45 to 45.66	31.64 to 37.69
Median	42.0	43.0	41.0	35.0
95% CI for the median	37.27 to 46.58	38.00 to 47.72	35.68 to 46.86	29.68 to 38.00
Variance	17.25	28.11	28.52	15.50
Standard deviation	4.15	5.30	5.34	3.93
Relative standard deviation	0.09 (9.97%)	0.12 (12.66%)	0.12 (12.85%)	0.11 (11.36%)
Standard error of the Mean	1.38	1.76	1.78	1.31
Coefficient of	−0.22	−0.19	−0.74	−0.81
Skewness	(p = 0.751)	(p = 0.776)	(p = 0.290)	(p = 0.246)
Coefficient of	−0.83	−0.83	−0.46	−0.51
Kurtosis	(p = 0.451)	(p = 0.449)	(p = 0.604)	(p = 0.580)
Kolmogorov-Smirnov test for	accept normality	accept normality	accept normality	accept normality
Normal distribution	(p = 0.9983)	(p = 0.9762)	(p = 0.8850)	(p = 0.7690)

Pearson correlation coefficients calculated between groups for the average number of electrical stimulations necessary for the development of full kindling epilepsy were not statistically significant. 

However, through application of the t test, we found that the number of electro-stimulations necessary for the development of full kindling was statistically lower in group E *versus* groups C, W and B (Table 8). 

**Table 8:** Statistical difference between mean values of the average number of electro-stimulations necessary for the development of kindling threshold after-discharge *.

		C	W	B	E
**C**	Difference		0.22	−0.11	−7.00****
	Standard Error		2.25	2.26	1.91
	95% CI of difference		−4.562 to 5.007	−4.918 to 4.696	−11.043 to −2.95
	Test statistic t		0.0990	−0.0493	−3.670
	Significance Level P		p = 0.9225	p = 0.9614	**p = 0.0021**
**W**	Difference	0.22		−0.33	−7.22****
	Standard Error	2.25		2.51	2.20
	95% CI of difference	−4.562 to 5.007		−5.65 to 4.98	−11.91 to −2.53
	Test statistic t	0.0990		−0.133	−3.281
	Significance Level P	p = 0.9225		p = 0.8959	**p = 0.0051**
**B**	Difference	−0.11	−0.33		−6.89****
	Standard Error	2.26	2.51		2.21
	95% CI of difference	−4.918 to 4.696	−5.65 to 4.98		−11.603 to −2.17
	Test statistic t	−0.0493	−0.133		−3.115
	Significance Level P	p = 0.9614	p = 0.895		**p = 0.0071**
**E**	Difference	7.00****	−7.22	−6.89****	
	Standard Error	1.91	2.20	2.21	
	95% CI of difference	−11.04 to −2.95	−11.91 to −2.53	−11.60 to −2.174	
	Test statistic t	−3.670	−3.281	−3.115	
	Significance Level P	**p = 0.0021**	p **= 0.0051**	**p = 0.0071**	

* using an independent samples t-test and assuming unequal variances.

**Figure 1:**
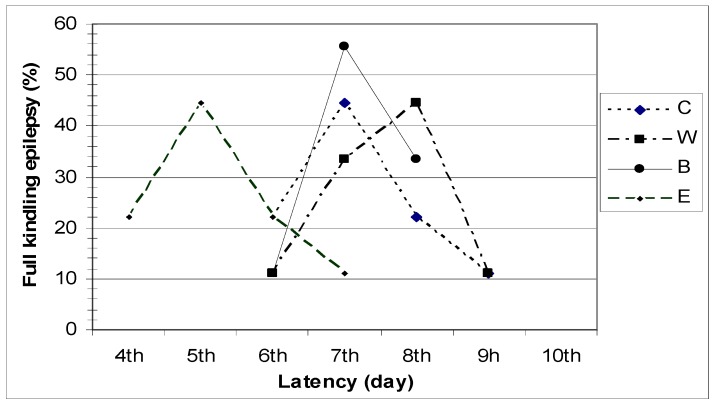
Latency period for the development of full kindling epilepsy.

As shown in Figure 1, the latency period observed before the development of full kindling differed in animals from different groups.

Based on Racine's seizure classification, two consecutive grade 5 seizures were observed in 44.44% of control group (C) animals by the end of the 7th electro-stimulation day, and in 22.22% on the 6th and 8th day of hippocampus stimulation.

In group W, the latency period before the development of full kindling epilepsy was eight days in 44.44% of the animals, seven days in 33.33%, nine days in 11.11%, and six days in 11.11% of the animals. 

In group B, the latency period before the development of full kindling epilepsy lasted six days in 11.11% of the animals, seven days in 55.55%, and eight days in 33.33% of the animals.

In group E, full kindling epilepsy was observed in 44.44% of the animals on the 5th day of hippocampal electro-stimulation, in 11.11% on the 7th day, and in 22.22% of the animals on the 4th and 6th day of stimulation.

Our results clearly show that application of the tested *Hypericum perforatum* L. extracts significantly influences the development of epileptogenesis and the development of full kindling epilepsy. 

With respect to the intensity of electrical stimulation necessary for the development of AD discharge, we observed that threshold currents necessary for the emergence of epileptic discharge were lower in control animals then in animals administered either aqueous or butanol extracts of *Hypericum perforatum* L. In fact, this difference was statistically significant between groups C and W, suggesting that administration of the water extract of *Hypericum perforatum* L. significantly inhibits the development of epileptic discharge. In contrast, treatment of animals with the ether extract of *Hypericum perforatum* L. lowered the minimum current strength necessary for discharge; the mean values of current intensity in this group were significantly lower *versus* the mean intensity observed for the control group.

In addition to the above, the influence of the tested *Hypericum perforatum* L. extracts on epileptogenesis is also supported by results from our analysis of AD duration. In animals administered ether extracts, AD durations were statistically significantly longer than controls, while animals treated with water or butanol extracts displayed shorter AD duration times than control animals.

Interestingly, treatment of animals with the ether extract of *Hypericum perforatum* L*.* had the opposite effect on epileptogenesis versus water and butanol extract treatments. In fact, the above analysis suggests that etheric *Hypericum perforatum* L. extracts may actually have epileptic effects. This negative influence from the ether extract of *Hypericum perforatum* L. was also observed in analyses of the number of electrical stimulations and the duration of the latency delivery period required for the development of full kindling epilepsy. In total, these results suggest that water and butanol extracts of *Hypericum perforatum* L*.* reduce the excitability of neurons, while, in contrast, the ether extract increases neuronal excitability. In addition, our results clearly show that the effect of Hypericum *perforatum L.* on epileptogensis strongly depends on the type of plant extract applied.

In agreement with the results of the present study, we have previously shown that administration of different extracts of *Hypericum perforatum* L. to experimental animals with already formed epilepsy has a similar effect on epileptic discharge: the non-polar ether fraction potentiates epileptic activity, while the polar fractions repress epileptic activity [26,27]. Similarly, in a study comparing the effects of ethanol, ethyl acetate, and water extracts of *Hypericum perforatum* L., extract polarity has been shown to affect neuronal excitability [28]. Moreover, the less polar extract fraction displayed significantly stronger analgesic activity [28].

According to Hosseinzadeh *et al.* [29], ethanol and water extracts of the aerial part of *Hypericum perforatum* L*.* increased the latency of convulsions induced by pentylenetetrazole in a dose-dependent manner in a mouse model of petit mal epilepsy. In addition, both extracts increased the latency of convulsions induced by an alternating current stimulus. The authors suggest that it is possible that this anticonvulsant activity may be mediated through the nitric oxide pathway.

Based on the results of the present study, it is clear that the different extracts of St. John's wort investigated here contain different active principles, which have different activities against the processes of epileptogenesis. To date, the known active constituents of St. John's wort are napthodianthrones (hypericin, pseudohypericin), flavonoids (quercetin, amentoflavone, hyperin), phloroglucinols (hyperforin, adhyperforin) and essential oils [14,15,16,17,30]. The concentration and composition of active constituents in the extracts tested here are different from case to case. 

Interestingly, it may be possible that the same active (constituents) principles of *Hypericum perforatum* L*.* that have been shown to have antidepressant effects may also have pro-epileptic effects. As demonstrated in numerous works, the antidepressant activity of hypericum extracts has been attributed to the phloroglucinol derivative of hyperforin, hypericin, as well as to napthodianthrones and pseudohypericin [18,19,31,32], which acts on transmitter systems. Initial biochemical studies reported that St John's wort is only a weak inhibitor of monoamine oxidase-A and -B activity. Hyperforin has been reported to inhibit the reuptake of a variety of neurotransmitters, such as γ-aminobutyric acid (GABA), 5-hydroxytryptamine (5-HT), dopamine (DA) and noradrenalin [33,34]. However, other *in vitro* binding assays carried out using St John's wort extract demonstrated significant affinity for adenosine, GABA A, GABA B and glutamate receptors. *In vivo*, St John's wort extract leads to down-regulation of β-adrenergic receptors and up-regulation of serotonin 5-HT2 receptors in the rat frontal cortex, and causes changes in neurotransmitter concentrations in brain areas that are implicated in depression [17]. These data clearly show that long-term, but not short-term administration of St. John’s wort and its active constituent hypericin modify the levels of neurotransmitters in brain regions involved in the pathophysiology of depression, the hippocampus and hypothalamus [35]. One of these brain structures, the hippocampus, is also responsible for initiation of epileptic discharge in our experimental model. We hypothesize that some active principles from the *Hypericum perforatum* L. extracts tested here stimulate GABA receptors, causing antiepileptic effects, while other constituents influence the level of catecholamines and act as pro-epileptics. However, it is necessary to further analyze the impact of specific active principles on epileptogenesis in different experimental models.

## 3. Experimental

### 3.1. Plant materials and Extract Preparation

Plant materials for all experiments were collected in eastern Serbia. Voucher specimens were taxonomically identified and deposited at the department of Botany at the University of Novi Sad. The plant material was air-dried, powdered and extracted with 70% ethanol in a Soxhlet apparatus for 12 h. Solvent was removed under reduced pressure. The residue was further partitioned by successive liquid-liquid extractions with water (W), *n*-butanol (B) and ether (E), evaporated to dryness, weighed, and finally dissolved in 50% ethanol to yield a mass concentration of 0.1g/mL. 

### 3.2. Biological Tests

Experiments were conducted on Chinchilla rabbits of both sexes. The study animals were obtained from a local conventional breeding colony. All study animals were housed in standard cages at 18 °C on a 12-h light/dark cycle (07:00 light on), had free access to water and food. Investigations were carried at the Laboratory for Neurophysiology at the Medical Faculty of Novi Sad. Study animals were divided into four groups, each consisting of nine animals (n = 9). The animals were treated as follows: (1) control group (C): solvent (50% ethanol); (2) group (W): water fraction of the crude *Hypericum* extract; (3) group (B): butanol fraction of the crude *Hypericum* extract; (4) group (E): ether fraction of the crude *Hypericum* extract. Extracts were administered beginning on the 60th day of life. The average bodyweight (BW) of the study animals at the time of administration was approximately 2 kg. Extracts were administered intramuscularly every day at 8 a.m., in a single dose of 1 mL/kg BW. Extracts (or solvent for control animals) were administered to experimental animals until the development of full kindling.

### 3.3. Research Procedure (Table 9)

**Table 9:** Research procedure.

Day after litter	60^th^	75^th^	80^th^	82^th^	90^th^
Procedure	Beginning of the investigation	Scalping	Electrode Implantation	Beginning electrostimulation of the hippocampus	Scarification
Extract obtained(in days)	1^th^	16^th^	21^th^	23^th^	31^th^

### 3.4. Scalping and Implantation

On day 75 (after birth), operational preparations were made for each animal for electrode implantation (on the 16th day after initiation of extract treatment). Rabbits were anesthetized with 10% novocaine and placed in a stereotaxic frame and positioned in a skull-flat position in asepsis and antisepsis conditions. The scalp was clipped and prepared with povidone-iodine before the surgical procedure. Six days later, electrode implantation was conducted (on the 21st day of extract administration). A sub-cortical electrode made of nickel chrome was isolated (except at the tip). A stainless steel stimulating/recording electrode (0.2 mm diameter) was placed stereotaxically into the dorsal hippocampus - in region Ca3. [36]. Electrodes were secured in place using acrylic dental cement. 

### 3.5. Formation of Epileptic Focus

Electro-stimulation of the hippocampus, and monitoring of bioelectrical activity was begun on the second day after electrode placement (on the 22^nd^ day of extract administration). For each experimental animal, the minimal (threshold) electric current intensity necessary to induce an after-discharge (AD) of at least 5 s duration was recorded. In order to determine the threshold current intensity, initial stimulation was conducted with 100 μA current, and was increased in 10 μA steps for each subsequent stimulation, until a threshold AD was observed. 

The initial minimum current intensity that caused an empty AD was considered to be the ‘threshold intensity’. After determining the minimum volume of electricity (the ‘threshold intensity’ necessary for the development of the epileptic discharge), minimum amperage (threshold) electrical stimulation was performed on the hippocampus.

Electro-stimulation of each rabbit was conducted by stimulation with an electrical train (50 Hz with a 1.5 ms square-wave for 1 s): one application consisted of 50 electrostimuli. The hippocampus was stimulated on the same side of the brain, and animals were stimulated six times daily >20 min apart from threshold stimulation. Electro-stimulation was repeated until the establishment of full kindling. A behavioral seizure score was analyzed after each kindling stimulation, using the Racine seizure classification scheme [24]. Animals were stimulated until they achieved two generalized tonic clonic activities with loss of posture in one day. That day was then used to define the latency period to develop fully generalized seizures.

The following parameters were determined for each animal: values of the minimum current strength necessary for initiation of threshold AD; duration of the threshold AD; number of electrostimulations necessary for the development of full kindling; and latency time for the development of full kindling (expressed in days). 

### 3.6. Histological Analysis

At the end of the study, all rabbits were sacrificed by intraperitoneal injection of pentobarbital (nembutal 150 mg/kg). The correctness of the electrode position in the hippocampus was determined histologically, and in case of any abnormality, data obtained from that particular animal was not included in the results.

All experiments were conducted in accordance with the internationally accepted Guidelines for accommodation and care of animals (European Council Directive of 24 November 1986, 86/609/EEC). 

### 3.7. Statistical Analysis

Results were analyzed using special “MedCalc” software. Results were considered to be statistically significant when p < 0.05. The effects induced by different extracts were compared using the Student t-test and by Pearson correlation.

## 4. Conclusions

In sum, our results show that water and butanol extracts of *Hypericum perforatum* L*.* reduce the excitability of neurons in a kindling model of epilepsy, and that an ether extract of *Hypericum perforatum* L. has pro-epileptic effects.

## Supplementary Materials

Supplementary File 1
