# Supplementary material for: Effects of St John's Wort (Hypericum perforatum L.) Extracts on Epileptogenesis
Source: Molecules. 2011 Sep 19;16(9):8062–75. doi: 10.3390/molecules16098062 (PMC6264782; doi:10.3390/molecules16098062)

Abstract: The purpose of this study was to investigate the effects of treatment with water, *n*-butanol and ether extracts of *Hypercom perforatum* L. on epileptogenesis in rabbits. Animals from the control group received solvent-ethanol, and the kindling model of epilepsy was used. Epileptic focus was induced in Chinchilla rabbits by stimulation of the hippocampus. The following parameters were determined: the minimum current strength necessary to induce after-discharge (AD) – discharges appearing after cessation of stimulation; AD duration; the number of stimulations necessary to induce spontaneous kindling; and the latency time for the development of full kindling. The results obtained indicate that epileptogenesis is influenced by *Hypericum perforatum* L. extract treatment. Animals treated with an ether extract of *Hypericum perforatum* L. required significantly weaker minimum current strengths for the development of epileptogenic focus, and displayed longer AD times, while the number of electro-stimulations necessary for full kindling was less. In contrast, animals treated with water and *n*-butanol extracts required increased electro-stimulations for the development of epileptic discharge, and displayed shortened AD durations *versus* controls.

Keywords: St. John's wort; *Hypericum perforatum* L.; extracts; kindling; epilepsy; rabbits

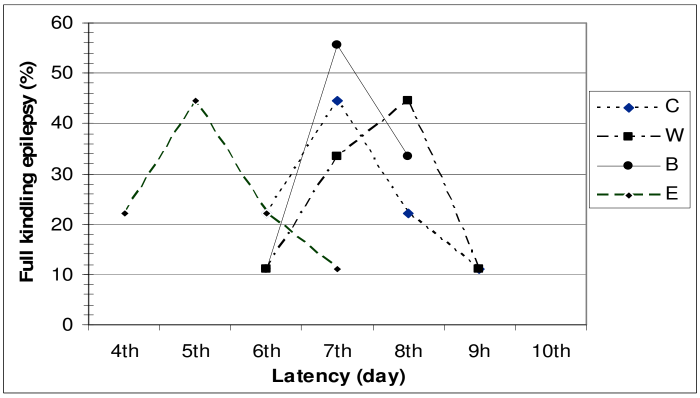

Supplement: Supplementary File 1 [file molecules-16-08062-s001.pdf]
